# Infants infer and predict coherent event interactions: Modeling cognitive development

**DOI:** 10.1371/journal.pone.0312532

**Published:** 2024-10-24

**Authors:** Johanna K. Theuer, Nadine N. Koch, Christian Gumbsch, Birgit Elsner, Martin V. Butz

**Affiliations:** 1 Neuro-Cognitive Modeling, Department of Computer Science and Department of Psychology, University of Tübingen, Tübingen, Germany; 2 Chair of Cognitive and Clinical Neuroscience, Faculty of Psychology, Technical University Dresden, Dresden, Germany; 3 Developmental Psychology, Faculty of Humanities, University of Potsdam, Potsdam, Germany; University of Giessen: Justus-Liebig-Universitat Giessen, GERMANY

## Abstract

Mental representations of the environment in infants are sparse and grow richer during their development. Anticipatory eye fixation studies show that infants aged around 7 months start to predict the goal of an observed action, e.g., an object targeted by a reaching hand. Interestingly, goal-predictive gaze shifts occur at an earlier age when the hand subsequently manipulates an object and later when an action is performed by an inanimate actor, e.g., a mechanical claw. We introduce CAPRI^2^ (Cognitive Action PRediction and Inference in Infants), a computational model that explains this development from a functional, algorithmic perspective. It is based on the theory that infants learn object files and events as they develop a physical reasoning system. In particular, CAPRI^2^ learns a generative event-predictive model, which it uses to both interpret sensory information and infer goal-directed behavior. When observing object interactions, CAPRI^2^ (i) interprets the unfolding interactions in terms of event-segmented dynamics, (ii) maximizes the coherence of its event interpretations, updating its internal estimates and (iii) chooses gaze behavior to minimize expected uncertainty. As a result, CAPRI^2^ mimics the developmental pathway of infants’ goal-predictive gaze behavior. Our modeling work suggests that the involved event-predictive representations, longer-term generative model learning, and shorter-term retrospective and active inference principles constitute fundamental building blocks for the effective development of goal-predictive capacities.

## Introduction

How do infants learn to understand their environment to flexibly act on and within it? Unlike adults, who have a rich tableau of experiences and previously built habits to draw on, infants have to learn how to interpret and interact with the world. In this, they are aided by so-called ‘core knowledge’ or ‘core principles’. For example, the concept of object permanence predicts that objects do not disappear if they are out of sight, while the principle of inertia predicts that objects will remain at rest or follow their current path as long as no additional external forces apply. Core knowledge is available from early infancy and constitutes fundamental building blocks for further cognitive development [1–4].

Infants’ mental representations of the environment are sparse at first, but as infants gain experiences from their sensory exploration and their motor interactions with the environment, their representations become richer and more complex over development [4–8]. Accordingly, there is converging evidence that infants represent observed interactions between objects as physical events in different categories, such as occlusion, containment, or collision events [8–13]. Infants’ developing understanding of knowledge about physical events can be explained by a two-systems model [4, 14]. Briefly, the model states that there is an *object-file system* that contains information about objects seen in the environment, especially about their features (e.g., shape, size, color) and category membership(s) (e.g., animate/inanimate object, animal/vehicle). Additionally, a *physical reasoning system* is involved when the infant observes or produces physical interactions between objects (one of which may be the infant’s or another person’s hand). The physical reasoning system draws on the object-file system for information about the involved objects, with the aim of interpreting and predicting the currently observed event. The interplay of the two systems can explain the emergence of errors in infants’ event interpretation, such as object individuation failures when infants have wrong (or no) expectations about the number of objects revealed at the end of an event [14, 15]. The characteristics of such failures also imply that infants draw upon different information, depending on the category of the current event (e.g., 3.5-month-olds regard an object’s height in occlusion events, but not yet in containment events) [4]. The infant’s two-systems model changes with age and is continuously refined by a process called *explanation-based learning*, in which infants validate, generalize, and differentiate their object files and physical event categorizations to account for observations that are inconsistent with model components [16].

Very early, infants begin to distinguish whether an object is human or non-human as well as whether an entity is self-propelled/animate or inanimate [4, 17, 18]. Based on studies measuring infants’ looking times to expected or unexpected events conducted by animate or inanimate objects, it has been suggested that these fundamental ontological distinctions may form the basis for learning more differentiated interpretations of observed object interactions [4, 19–23]. Additionally, apparent causal dependencies and visual cues provide important information for particular object or event categorizations. For instance, in object interaction events, infants see animate objects as potential actors and inanimate objects as potential patients [24], and they expect human figures and hands to act in an intentional, goal-directed manner [25, 26].

In relation to the explanation-based learning principle [16], it has been suggested that humans generally try to create *coherent interpretations* of observations. That is, humans use event-predictive models and evaluate whether their current observations match their predictions, striving for internal consistency in their interpretations of events and consistency with sensory input [27–32]. The active search for coherence has been shown, for example, with respect to predicting plausible and grammar-conforming input while reading, based on continuously updated referential models [33–35]. In the visual domain, human minds fill in missing information by means of backward, retrospective inference processes for coherent event interpretation [36]. Furthermore, implicit causal inferences influence the understanding of events as well as how event interpretations are segmented and stored into episodic memory, especially as part of narratives [32, 36–40]. We posit that infants also try to create coherent interpretations when observing events that involve interactions between objects. In particular, we propose that *retrospective inference processes*, which strive to establish coherence between internal interpretations and perceived observations, may help explain the development of anticipatory gaze behavior in infants.

When observing object interaction events, infants’ gaze behavior is strongly influenced by their action experience [41, 42]. Eye-tracking studies found that when observing a simple goal-directed action (such as a hand reaching for and grasping an object), infants from a certain age onward shift their gaze from the moving actor (i.e., a hand) to the goal object before the actor arrives there, predicting its arrival [43]. In contrast to reactive or tracking gaze (i.e., looking at the goal object only after or at the moment when the actor arrives there), predictive gaze shifts indicate that infants infer goals or intentions when observing action events. Interestingly, such goal-predictive gaze shifts emerge from about 7 months of age, starting with animate, familiar actors (i.e., human hands) and familiar actions (e.g., grasping). After a few more months of development, infants perform goal-predictive gaze shifts when observing the same actions performed by an inanimate actor (e.g., a mechanical claw) [29, 41, 42, 44–46]. Goal-predictive gaze behavior seems to depend on infants’ motor ability and their experience about interacting with an object, as well as on the appearance of an actor and the characteristics of the unfolding movement [29, 42, 45–47]. In particular, infants can build expectations about action outcomes or movement goals better for actions that they can already perform themselves and for agents that they have observed frequently, including their own and other persons’ hands [29, 46–48]. These observations support the view that infants build internal event-predictive models and use these models to interpret their observations. They also support the two-systems model [4], indicating that performing or observing object-directed actions or physical events that involve object interactions support the development of both the physical reasoning system and the object-related processes that enable the attribution of agentiveness.

**Fig 1:**
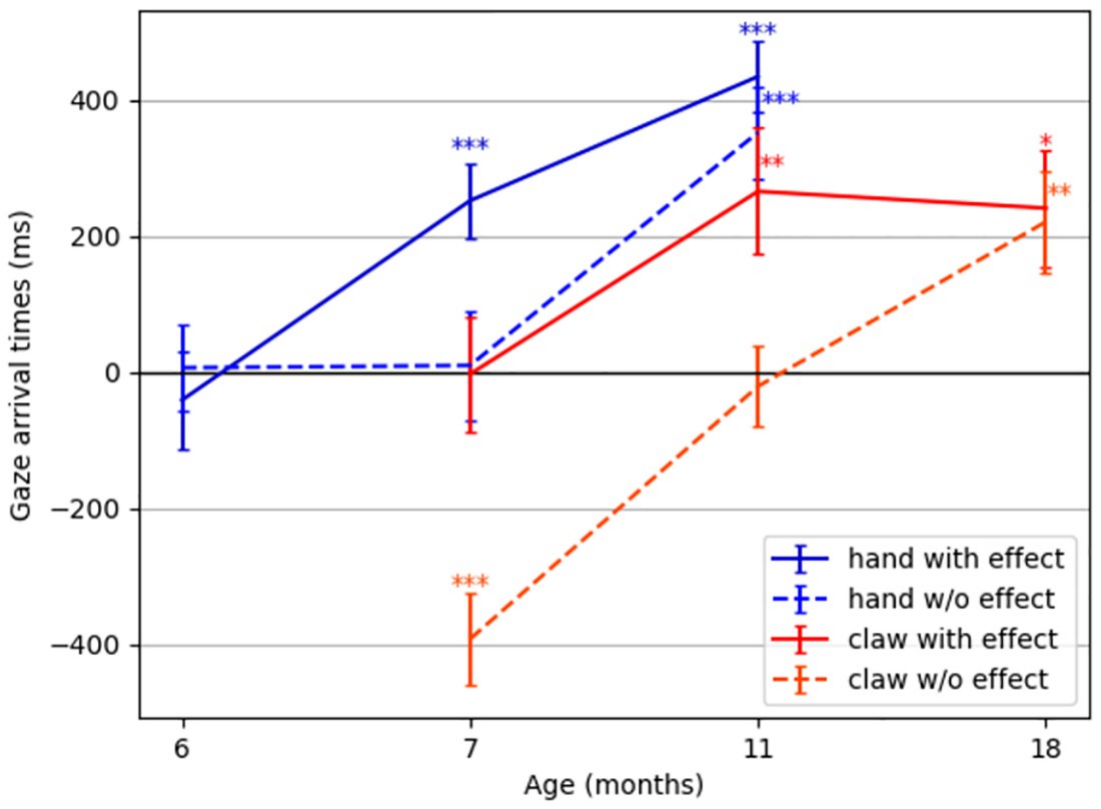
Infants’ gaze-arrival times. The difference in time from the arrival of the gaze at the goal object to the arrival of the actor (measured in ms), for infants at various ages watching videos of hands or mechanical claws grasping a goal object with or without producing an action effect (i.e., lifting the object after grasping it). Positive values indicate goal-predictive gaze shifts. Error bars show standard errors. Data is taken from several studies [44, 46]. Asterisks indicate a significant difference from 0 ms: *p<.05, **p<.01, ***p<.001.

A series of experiments in which infants who repeatedly watched a hand or a mechanical claw reaching for and grasping a goal object [43–45, 49] reveals an intriguing developmental pathway of infants’ goal-predictive gaze behavior. As summarized in Fig 1, both the type of actor as well as the presence of agency cues determined at which age infants started to predict the goal of the unfolding movement: First, at around 7 months, infants showed goal-predictive gaze shifts for hands, but only when a salient action effect followed the grasping movement, that is, when the hand lifted the goal object. For the same action and effect performed by a mechanical claw, predictive gaze shifts emerged only at around 11 months. At that age, infants also showed predictive gaze shifts for human grasping without an action effect (i.e., when the hand froze upon touching the goal object). Again, for a grasping mechanical claw without action effect, this occurred only later, at around 18 months. Infants tested at younger ages with the respective actors and actions mainly fixated the actor until it finally grasped the goal object. The parallel but age-shifted developmental patterns of predictive gaze behavior have been interpreted as relying on infants’ growing experience with performing and observing goal-directed actions of human hands and of actions that involve the use of tools such as spoons or sticks to retrieve objects [29].

### Modeling approach

We show that a self-developing two-systems model of the environment in interaction with inference processes, which are unfolding within the developing model, can explain the observed developmental patterns. Based on the previous CAPRI (Cognitive Action PRediction and Inference in Infants) model [50], we offer an implementation of these processes in a simple simulated scenario. Broadly, we posit that the infant’s two-systems model is implemented by means of object files, which include an estimation of agentiveness, and schematic event knowledge [51], which encodes how events start, unfold, and end. For example, the model may encode that a hand that touches an object may start lifting it, and the observation that a hand lifts an object may indicate an ongoing (agentive) transportation event. The model’s inference processes control (i) longer-term development of the world model, (ii) shorter-term event-predictive interpretations of sensory observations, (iii) retrospective interpretation adaptations to further enforce interpretation coherence, and (iv) predictive eye gaze behavior.

We believe that (i) is a slow learning process that relies on sufficient sensorimotor experiences to generalize event knowledge across situations and involved entities—e.g., that objects that do not look like hands may actively reach for objects. This slow model learning process might explain the difference in age between the occurrence of predictive gaze shifts for a familiar hand and an unfamiliar claw (Fig 1). In contrast, (ii) and (iii) quickly and temporarily adapt internal beliefs to maximize coherence between model predictions and observations. Event inference infers internal event-predictive model states that best explain the perceived observations, predicting the observation dynamics. Retrospective inference draws on the just interpreted event sequence and adapts the model’s currently active contextual representations and involved belief states, with the aim of maximizing the coherence of the model’s estimates with the encountered observations even further [52]. This process can, for example, lead to the reinterpretation of a mechanical claw from an inanimate to an animate agentive entity when it exhibits agency cues, such as when lifting an object [45]. As a result, beyond simply learning to recognize unfamiliar actors performing goal-directed reaching as time goes on, such short-term retrospective inference mechanisms may explain the earlier goal predictions for entities that perform (agentive) actions followed by salient effects (Fig 1).

#### Cognition as inference

Prominent theories of predictive processing view cognition and behavior as inference mechanisms [53–55]. This is particularly the case in Bayesian brain theories, which posit that the human mind heavily relies on estimated probabilities and Bayesian updating as inference [56, 57]. Computations in the brain update the distributions of belief states to explain perceived processes, potentially triggering motor actions. Our model assumes that interpreting an observed process and predicting its outcome critically hinges on *event-predictive inference* [28]. According to the theories of event coding [58, 59] and of event segmentation [60, 61], events are captured by representations with defined boundaries. These representations can be modeled as event codes used in computational cognitive models [28, 50, 59, 62, 63]. Our model implements event inference by iteratively updating representations and (prior/posterior) distributions of event-predictive and entity-encoding belief states. To support the inference processes, the model chooses gaze policies in order to gather epistemic information about the currently unfolding event and, given the currently inferred event estimates, about anticipated event transitions.

#### Modeling hypotheses

We thus hypothesize that the developmental patterns of goal predictions (Fig 1) can be fully explained by internal event inference, epistemic gaze selection and retrospective inference given the inferred events. In particular, we assume that infants segment their experience into events. These developing event codes are believed to schematically encode objects and their sensorimotor trajectories with defined boundaries, supporting the effective prediction of future sensory inputs [28, 51, 58, 61]. Based on a currently inferred probabilistic event density, infants can predict the unfolding movements and anticipate next potential event transitions. They thus become able to prepare processing for upcoming sensory information by directing their gaze toward locations where information gain about the unfolding event or anticipated transitions is expected to be maximal.

But how may this be accomplished algorithmically? We implemented four interwoven mechanisms that realize the described inference and control processes. First, model learning works slowly, over longer time scales, to update and expand the developing event-predictive models and improve their suitability for interpreting and navigating the environment [64]. Second, when the model observes concrete environmental interaction dynamics via simulated sensory information (see Section Simulation Setup in CAPRI^2^ for a description), event inference unfolds. In particular, the model interprets the available sensory information by means of its object files and event-predictive model. It thus infers event interpretations (e.g., reach, transport) that best explain the sensory observations. Third, retrospective inference draws on events that have just passed and adjusts the inferred probabilistic event densities and object file estimates to further maximize interpretation coherence [52]. Finally, active inference is used for action selection (here, gaze control), prioritizing the minimization of expected free energy [65–67]. In the modeled scenario, this corresponds to minimizing belief uncertainty, that is, maximizing anticipated information gain [66, 67], given the current observation.

#### CAPRI^2^

**Fig 2:**
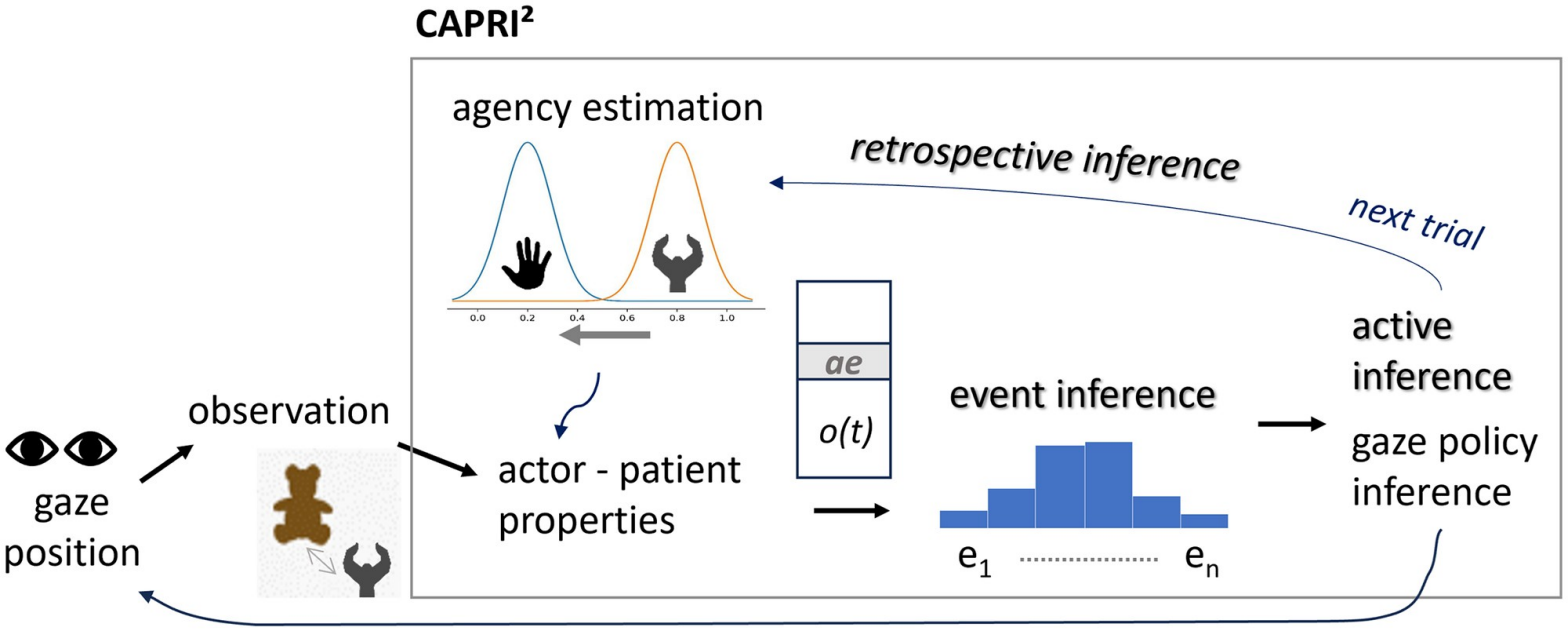
Schematic representation of the CAPRI^2^ model. Observations of the actor and patient, as well as the gaze policy, are used as input for the model, while the observation is affected by a noise level dependent on the chosen gaze position. Event probability distributions (for four events and three components per event type) are encoded in neural networks modeling probability distributions over observations and gaze policies. The agency estimation may be adapted internally by the retrospective inference mechanism to maximize interpretation coherence for the following trial. Given the resulting, potentially internally adapted, observation vector *o*(*t*), event inference adjusts the event likelihoods of the model, expressed in the encoded event probability distributions. The gaze policy (i.e., looking at the actor, patient, or elsewhere) is then chosen by active inference to minimize expected free energy.

We implemented the modeling hypotheses in a computational model, which we term CAPRI^2^ (Fig 2). CAPRI [50], as a model for infants’ goal-predictive gaze shifts, models experience-based event processing by learning likelihood distributions for the typical start and end conditions and the dynamics of an event in a probabilistic manner (schematic event encodings; see Section Materials and methods). At each time step, these encodings are used to make predictions about the next time step based on the current observation and gaze policy. Further, CAPRI returns probabilities for currently observing each learned event and chooses the gaze policy that minimizes the expected future uncertainty [50]. CAPRI^2^ enhances CAPRI by introducing longer and more diverse model learning and, crucially, an additional retrospective inference process that optimizes interpretation coherence by adapting object file representations and event probability densities. The densities that encode the start condition, the dynamics, and the end condition of event-predictive schemata are encoded by neural network models. Eye gaze behavior (e.g., looking at the actor or goal object) is controlled by active inference processes given the current model’s belief state. In addition to these processes guiding the model’s inference and gaze behavior for every time step within each trial, the retrospective inference mechanism updates an actor-specific agency estimate across trials. To the best of our knowledge, CAPRI^2^ is the first model that is able to computationally explain how infants’ goal-predictive gaze shifts may develop, offering concrete algorithmic hypotheses about critical underlying mental representations, cognitive structures, and inference processes.

#### Simulation and model training

CAPRI^2^ uses a simulation of a simple interaction scenario with one actor and a goal object (hereafter referred to as patient). Information about these entities, namely their positions, velocities, and distances, are used by the model as sensory input in an observation vector *o*(*t*). Also included are prior agency estimates for the actor and the patient, both ∈ [0, 1]. We treat a value of 0.2 as a canonical hand and a value of 0.8 as a canonical claw/non-agentive entity. During model training, the simulation generates four events: *e*_reach_ (i.e., the actor approaches and finally grasps the patient), *e*_transport_ (i.e., actor and patient move together), *e*_random_ (i.e., the actor moves in a random direction while the patient stands still), and *e*_still_ (i.e., both objects stand still). We trained our model with full grasping sequences, where the actor performs a reach, transport, and final random motion event, and with random motion events ending in a standing still event. We tested the model on full grasping sequences (*with an action effect*) in addition to simple reach events (*without action effect*), ending when the actor had grasped the patient.

Across 30 phases with 100 event sequences each, model training simulated infant development by means of longer-term inference. To keep things tractable, the model was trained in a supervised manner. That is, during training (but not testing), CAPRI^2^ was informed about the type of event that was currently unfolding. In particular, the model learned to predict the event-characteristic observations when an event starts, unfolds, and ends using several neural networks. To account for infants’ progressively more complex object interactions and object manipulation competencies [68], including the manipulation of other objects by means of tools (such as spoons or sticks), we trained the model on reach and transport actions executed by actors that increasingly varied in shape between the typical hand or claw/non-agentive entity (see Section Simulation Setup in CAPRI^2^ for further details).

After each of the 30 training phases, the model was evaluated in test trials. Each test trial showed either a full grasping sequence with action effect (i.e., *e*_reach_ → *e*_transport_ → *e*_random_) or only reaching without an action effect (*e*_reach_ only). 12 test trials each were run for actors with different shape parameters, which varied between 0 ‘hand-like’ and 1 ‘claw-like’ in steps of 0.1.

#### Short-term event inference

For each time step, the model receives an observation vector *o*(*t*), updates its internal object file representation, and interprets the observation by adjusting its internal event schematic densities. The inferred event probabilities for *e*_still_, *e*_random_, *e*_reach_, and *e*_transport_ were critical to the model’s performance during testing. The model also chooses its current eye gaze (on the actor, patient, or elsewhere) by means of its gaze policy. The model’s object file representation includes an agency estimate, which is important for our variation of the test trials. The interpreted observation and current gaze are fed into the event-schematic networks to predict the next observation in the form of a multivariate Gaussian density. Given the next observation, the event interpretation densities can be updated by maximizing the log-likelihood of the model’s probabilistic event estimates. This probabilistic inference mechanism thus interprets the interactions between the actor and the patient in an event-oriented manner. The model’s processing dynamics are depicted in Fig 2.

#### Active gaze inference

The event-structured likelihood distributions encoded by the model are also used to calculate the expected free energy for simulated eye gaze behavior, i.e., looking at the actor, the patient, or elsewhere. According to the principles of active inference [55, 66], the gaze policy with the smallest expected free energy is executed. Because the expected observations and thus expected free energy depend on the currently inferred event probabilities, simulated gaze behavior intricately depends on the event-predictive model structures learned so far as well as on the short-term inference processes concerning events and agency. The time point at which the model chooses to look at the patient for the first time was the critical measure in the test trials.

#### Short-term agency inference

As described, infants start to predict action goals earlier for familiar agents and for actors that produce action effects than for unfamiliar actors that do not display agency cues [43, 45, 49, 69, 70]. We propose that the creation of coherence influences infants’ agency estimations. CAPRI^2^ updates its agency estimation across the 12 test trials to increase its interpretation coherence of observed interaction sequences retrospectively at the end of each trial. At first, the object file-specific agency estimate is set directly to the prior agency estimation value indicated by the sampled shape of the entity (e.g., 0.2 for a canonical hand and 0.8 for a claw). After each observation of an event or event sequence showing the identical actor, this estimate is adapted to further improve trial interpretation coherence. This is assessed via the log-likelihood the model ascribes given the sensory data of a whole observed interaction sequence including the inferred event density dynamics (see Section Optimizing Coherence). In this way, coherence between the model and its interpretations (i.e., its internal event likelihood distributions) and the current observations and gaze policy is maximized.

## Results

**Fig 3:**
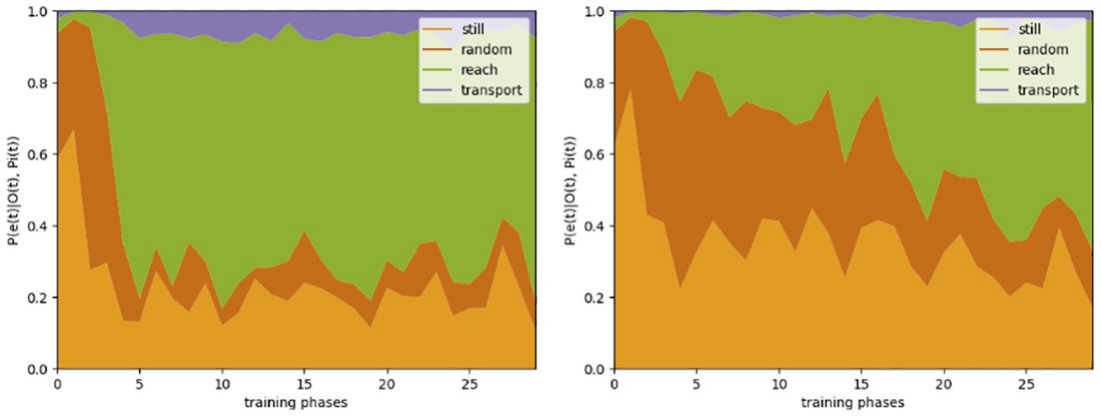
CAPRI^2^’s estimated event probabilities. The mean inferred probabilities for four events depending on the training phases of the model, assessed during the reach event of a grasping sequence, for a hand (shape 0.2; left) and a claw (shape 0.8; right).

**Fig 4:**
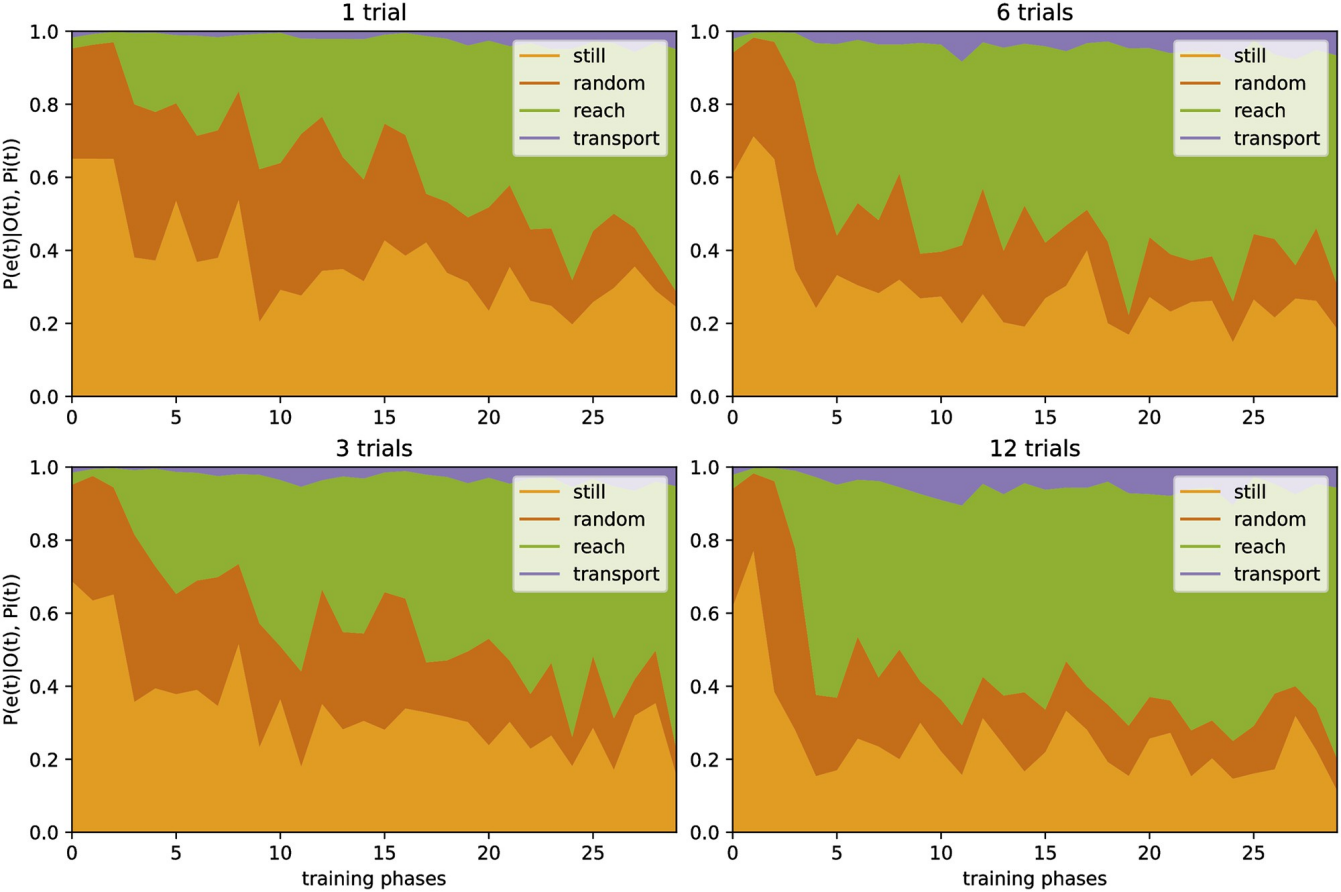
Influence of the retrospective inference mechanism on CAPRI^2^’s estimated event probabilities. The mean inferred probabilities for four events depending on the training phases of the model, assessed during the reach event of a grasping sequence for a claw (shape 0.8), after the coherence optimization mechanism had adapted the internal agency estimate for 1, 3, 6, or 12 test trials (top to bottom and left to right).

**Fig 5:**
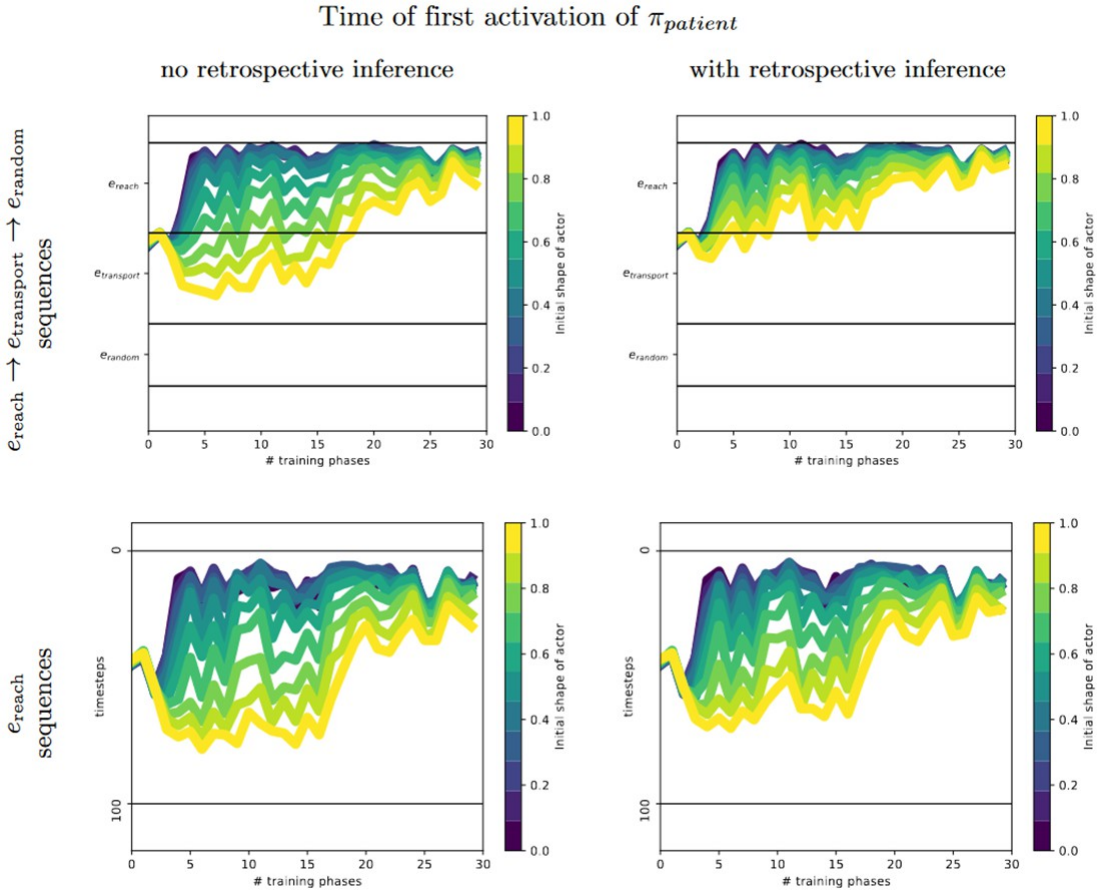
CAPRI^2^’s first gaze at the patient with (top) and without (bottom) action effects, by evaluating its behavior on *e*_reach_ → *e*_transport_ → *e*_random_ sequences (top) or *e*_reach_ sequences (bottom). The left and right column contrasts the resulting gaze behavior when retrospective inference (i.e., coherence optimization of the internal agency estimate) is not applied (left) or is applied (right). The y-axis plots the first gaze at the patient/goal during test trials, where the time sequence moves from top to bottom. The test trials were interleaved with the training phases (x-axis) to visualize the effect of model training on behavior. Higher y-axis values indicate earlier gaze shifts to the patient/goal, being predictive when occurring during *e*_reach_. Colored lines indicate the tested actor shapes: from dark blue ‘hand-like’ to yellow ‘claw-like’.

**Fig 6:**
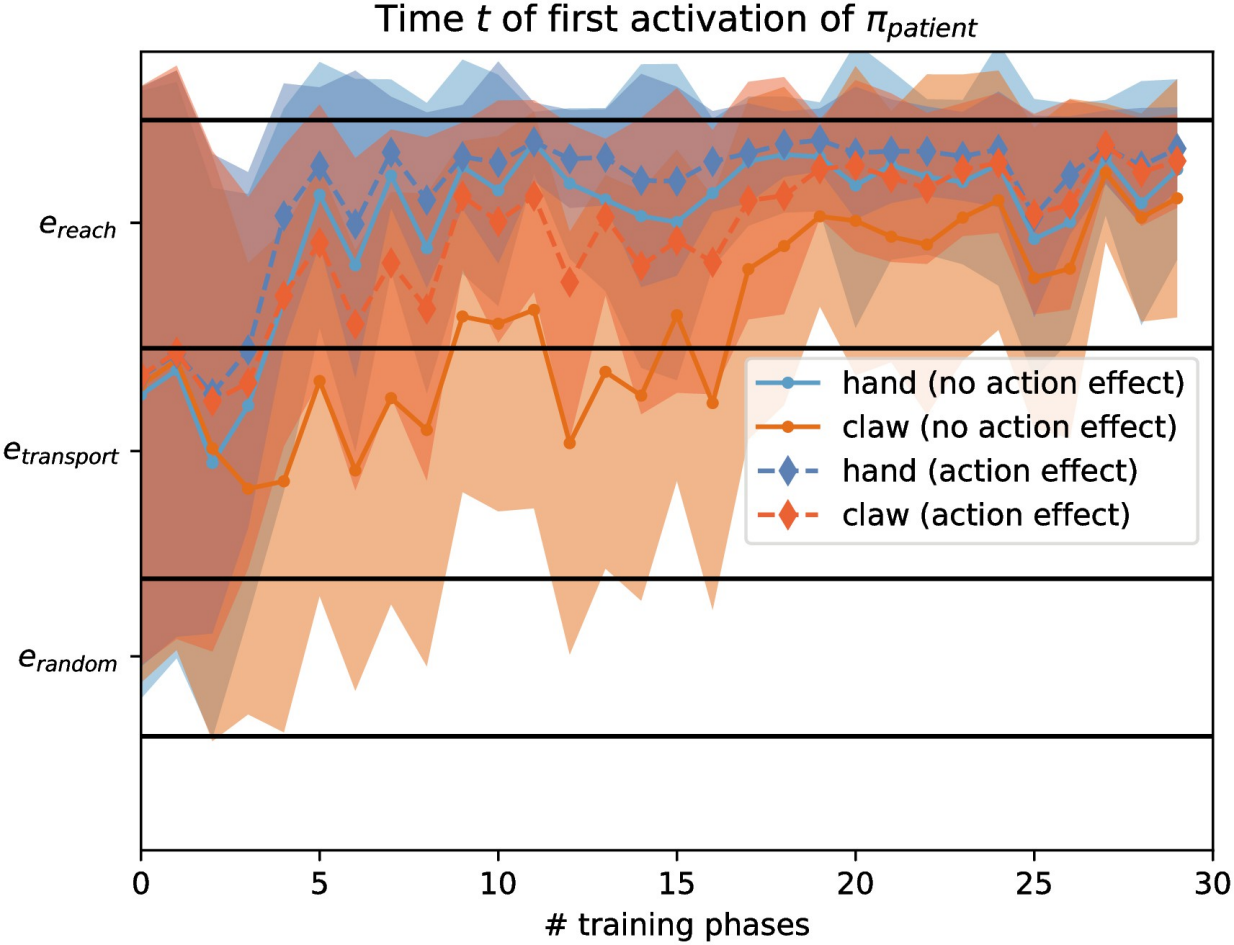
Comparison of CAPRI^2^’s gaze arrival times for hand-like actors (shape 0.4; blue) and claws (shape 0.8; red), when tested on full grasping sequences with action effect (dashed lines) or on reach-only events without action effect (solid lines), depending on the training phases (x-axis). Higher y-axis values indicate earlier gaze shifts to the patient/goal, being predictive when occurring during *e*_reach_. Shaded areas depict standard deviations.

**Fig 7:**
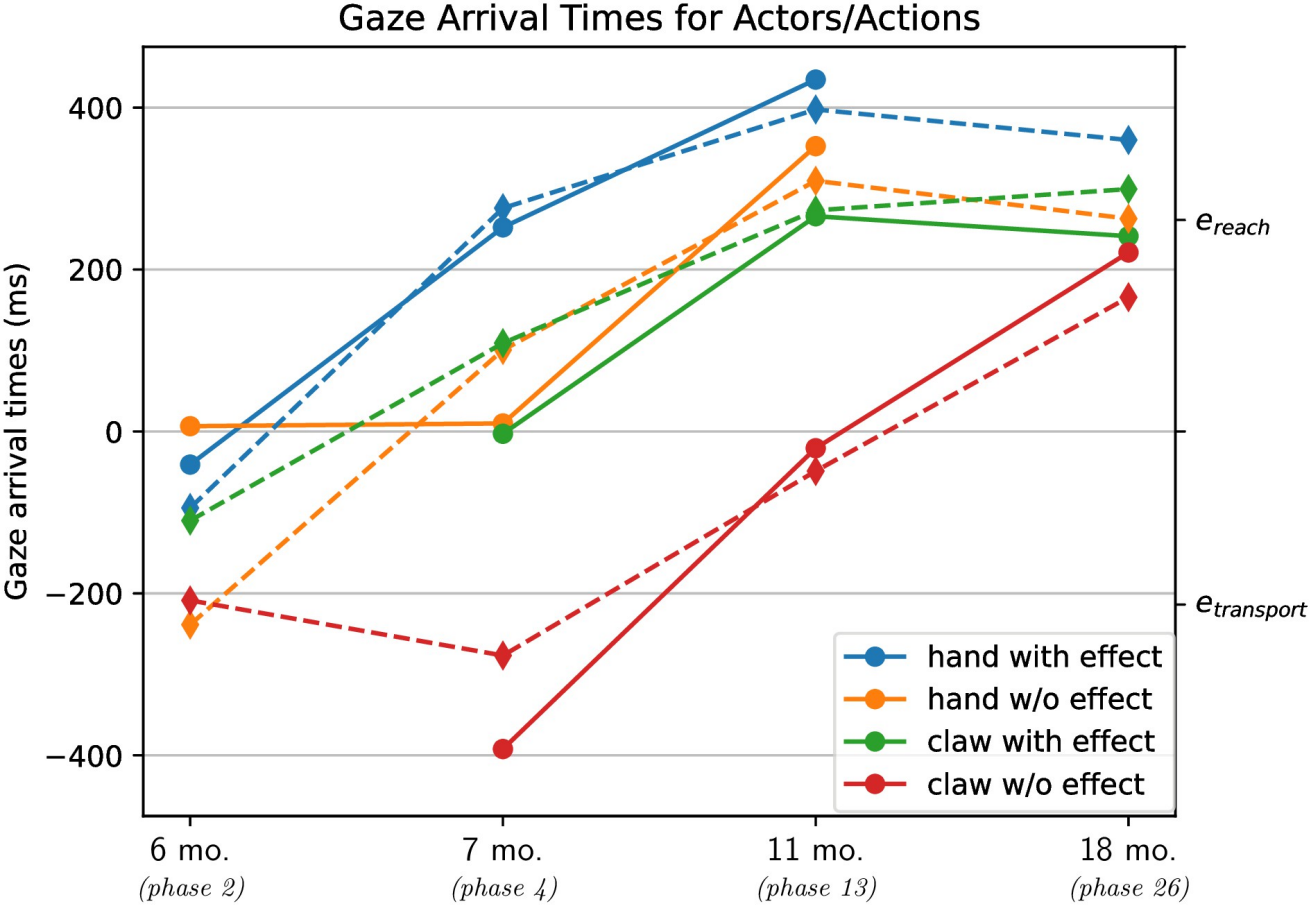
Comparison of infant and model data. CAPRI^2^’s gaze arrival times (dashed lines) for hands (shape 0.4) and claws (shape 0.8) with or without action effects after 2, 4, 13, 26 training phases, and infants’ gaze-arrival times (solid lines) at ages 6, 7.5, 11, 18 months.

To evaluate our model and its simulation of infants’ eye gaze development, we interleaved *training phases*, in which the model was trained in a supervised manner based on a number of event sequences, with *testing phases*, in which we mimicked the experimental conditions of developmental studies investigating infants’ predictive gaze behavior (e.g., [45]). We trained the model for 30 training phases, showing full grasping sequences (largely performed by hands but with increasing variance in their shapes), random motion sequences (performed by both hands and claws equally), and standing-still events. Testing was conducted in various conditions: with or without the coherence optimization mechanism to adapt the agency estimate, and with either full grasping sequences *with action effect* that consisted of the events *e*_reach_, *e*_transport_, *e*_random_, or reaching *without action effect* that consisted of only a *e*_reach_ event that ended with the actor grasping the patient. Figs 3–7 report the mean results for 20 independent model training and testing simulations.

The event probability estimates of the model show the expected pattern, similar to previous work [50]. For grasping movements performed by a canonical hand (shape 0.2), the model quickly learned to identify *e*_reach_ correctly, even without agency estimate adaptation (see Fig 3). For a canonical claw (shape 0.8), the model first predicted mostly the events *e*_random_ or *e*_still_. In later test phases, however, the model inferred a progressively higher probability of *e*_reach_, as later training phases included more diverse actors reaching for objects. With the agency estimate being adapted for a successive number of trials, the model’s inferred event probabilities showed an earlier recognition of *e*_reach_ performed by a claw and a similar pattern as for a hand (see Fig 4).

When analyzing the modeled gaze behavior, we found that the coherence optimization mechanism resulted in a qualitatively similar pattern as found for infants’ goal-predictive gaze shifts. The mechanism helped the model to infer earlier that claw-like entities may act agentively. As a result, the model began to identify the observed motion of more claw-like looking entities (actors’ shape closer to 0.8) as a *e*_reach_ event earlier. The model also chose the gaze policy to look at the patient (instead of the actor), resulting in predictive patient gazes already during reaching, earlier during learning compared to trials without coherence optimization (see Fig 5).

We also compared the model’s gaze shifts over the course of the test trials for full grasping sequences with an action effect (i.e., *e*_reach_, *e*_transport_, *e*_random_), and for only *e*_reach_ events without an action effect (Fig 5). As expected, CAPRI^2^ produced the general pattern of earlier predictive gaze towards the patient when watching a hand-like actor (shape = 0.4, modeling something like the hand of a stranger) and later when watching a claw-like actor (shape value near 0.8). At the same time, the model produced predictive gaze shifts to the patient after fewer training phases when observing sequences with as compared to without action effects (Fig 6). This qualitatively reproduced the pattern observed in infants when they watch grasping actions conducted by hands or mechanical claws with or without action effects (as shown in Fig 7).

## Discussion

Infants become able to predict the goal of a movement [29, 43–45, 47, 49, 69–74]. This ability is influenced by the familiarity of the movement and actor and by agency cues such as the production of action effects [29, 43–45, 49, 69, 70, 73]. Infants around the age of 7 months show goal-predictive gaze shifts for grasping movements by familiar actors, such as hands, and tracking gaze for unfamiliar actors, such as mechanical claws [29, 43, 45]. Some months later, infants also show predictive gaze when observing grasping performed by a mechanical claw or when observing only the reach, without a subsequent action effect [29, 43, 45, 69]. Our results indicate that the event-segmentation theory [60, 61], theories of predictive coding [53, 54, 57], and the free energy principle [55] provide the theoretical basis for explaining infants’ goal prediction with a cognitive model.

CAPRI is such a model for infants’ goal-predictive gaze shifts [50], based on probabilistic event schemata supporting inference processes and choosing gaze policies with active inference. CAPRI produced similar gaze behavior as infants at a certain age [41, 46] when observing grasping movements conducted by a hand or a mechanical claw [50]. This corresponded to the gaze behavior of 7-month-olds as depicted in Fig 1 [43, 45].

Here, we developed CAPRI^2^ to model infants’ developmental pathway of eye gaze behavior as found in [43, 45] (cf. Fig 1): Around the age of 7 months for hands and 11 months for mechanical claws, infants predicted the goal of grasping movements only when an action effect was observable. As research has found that humans try to create coherence in their perception [33–36], we hypothesized that infants also try to infer coherent interpretations.

We assumed that agency cues influence the interpretation of a movement. Infants would update their beliefs about the current events and contextual belief state components, such as their agency estimation of the actor, to increase the coherence of their perception and expectations. Hence, CAPRI^2^ incorporates a mechanism to increase the coherence of its interpretation of an observed interaction. In the addressed simulations, this mechanism led to the adaptation of the agency estimation of the actor (which is initialized with values that are based on the actor’s shape). As such, by using the model’s (log-)likelihood of the sequence (how likely is the current observation, given the currently inferred event estimates from previous observations) to adapt internal environmental interpretations, the model tightly links its own beliefs with the input for its interpretations. Accordingly, in the simulations, the adaptation of the agency estimate of the actor was central for enabling the model to interpret a reach event that is conducted by a visually appearing inanimate object correctly. The required agency estimates were thereby derived from the observation of a subsequent ‘transport’ action, which is unambiguous in its dynamics and is believed to be only executable by an agent.

Additionally, CAPRI^2^’s training scheme is designed to enable learning of the ‘agentiveness’ of previously unfamiliar actors (claws) when they are seen to perform grasping sequences that include an action effect of lifting the grasped object. Previously, CAPRI did not allow for this, only showing predictive gaze shifts and properly identifying reach events for hands, not claws. CAPRI^2^’s event probabilities showed the expected pattern, quickly learning to recognize reach events for hands, while only slowly differentiating between random motion and reach events for similar movements executed by claws (Fig 3). Simultaneously, CAPRI^2^ showed goal-predictive gaze: it looked at the patient before the actor grasped it, as this minimizes future uncertainty. This process occurred much quicker with the help of the retrospective inference mechanism (Fig 5). The resulting adaptation of the agency estimate is a key part of CAPRI^2^ and crucial for interpreting the input as a reach event. As a result, the predictive gaze shifts indeed occurred earlier when the model saw a full grasping sequence with action effect, compared to viewing only a reach event without lifting the target object (Fig 6).

CAPRI^2^ thus shows that event-based models can account for not only the goal prediction of infants but also the developmental patterns. To succeed, the model had to distinguish between agentive and non-agentive entities by means of an internal agency estimate. Further, it had to adapt this estimate retrospectively across trials by optimizing the coherence of its interpretations. It is important that these distinctions are not static but can change over development as infants learn. This holds at the level of individual event sequences, where infants, like adults, strive for coherence and adapt their estimation or belief states, and also in the longer term, when infants learn to distinguish objects or to use tools and expand their physical reasoning abilities [5, 16, 29, 68]. Accordingly, throughout training, CAPRI^2^ learns that even mechanical claws can generate grasping events, which reflects infants’ learning after they gain new experiences and are exposed to a growing variety of actors and events.

Importantly, while this learning was possible by using a training scheme that gave CAPRI^2^ simply ‘experience’ with previously apparently ‘non-agentive’ actors performing grasping sequences with an action effect, this learning took considerably longer without the retrospective inference mechanism. The presence of agency cues helped the model to disambiguate the actor’s apparent agency across several training phases, mirroring the pattern of development of infants’ goal-predictive gaze shifts (Fig 7). This argues for the drive to maximize coherence with retrospective inference as an important component in infants’ cognitive development of goal prediction, beyond other explanations, such as simply learning to recognize unfamiliar actors or increased cognitive capacities.

CAPRI^2^ also gives insights into infants’ potential mental representations. The fundamental distinction between agents and other objects, as well as how it can be bridged and expanded, becomes apparent. Recent theories such as the two-systems model [4] describe object files and event categories as fundamental for infants’ physical reasoning and their understanding of the world. While CAPRI^2^’s specific implementation of a neural network-based event model does not aim at mimicking neurobiological implementations in humans, it shows, as a proof-of-principle, how the two-systems model may be implemented. Clearly, the model could be extended with more event codes and with mechanisms to generate its own event codes. The number of events and order of sequences are fixed for now, and the agency estimate is added into CAPRI’s existing structure. Further, individual object files are implicitly assumed and enforced, given the structure of the simulation and the presented observations. Thus, with regard to the two-systems model [4], CAPRI^2^ focuses more on physical reasoning than on object representations. Yet, the interplay of the two systems is nevertheless visible in the introduced retrospective inference mechanism that estimates the actor’s agency driven by the aim of optimizing interpretation coherence. In sum, the modeling success suggests that the inference processes, based on categorical event codes and experience with actors of various shapes, capture key characteristics of infants’ physical reasoning, both in the theory and in the model implementation.

Our model can be linked to recent work in infants’ action understanding, especially a review and mega-analysis [75]. While focusing on younger, pre-reaching infants and on tasks requiring either the individuation of two goals or the representation of constraints hindering the goal approach, the authors emphasize the critical role of observable action effects and concepts such as ‘goal’, ‘cause’, and ‘cost’ in the development of infants’ understanding of goal-directed actions. This corresponds to our current setup, where the actor’s shape and the presence of agency cues act as strong information, enabling the model to infer an agentive, goal-directed action. Extending CAPRI^2^ to model these or similar results suggests itself as future work. For instance, probing the model’s agency representation against infants’ looking times in violation-of-expectation paradigms, i.e. by adding implausible changes to previously observed event sequences, could lead to interesting insights.

It is also important to note that event schemata do not operate in a vacuum. Rather, they are informed by and linked with the context in which they occur. In our simulations, the number of events and order of sequences are predefined, but the context of the events shown to the model differs: Either an actor actively interacts with the patient (i.e., reach, transport) or two mechanical objects appear to move according to physical laws (i.e., random motion). Notably, these observations would belong to different event categories [4] or contexts. This fundamental distinction feasibly calls for different contextual representations, where an agent—in contrast to an inanimate object—may pre-activate different options of event interpretations. The realization of such more explicit context dependencies may be taken into account for further model expansions (to compare, see also the COIN model [76] and its recent application to motor control [77]). Another open challenge is a model based on CAPRI^2^ that could learn to form new event structures. This could offer insights into not just action understanding in infants but, more broadly, event-based cognition and its development. Regarding context differences while learning and interpretation events, it may further be noted that CAPRI^2^ incorporates both ‘apparent learning’ and ‘proper learning’ [78]. Contextual codes and cues, such as the actor’s shape, lead to a change in event code access. While the adaptation of the agency estimate and the general process of event inference corresponds to apparent learning, the model’s learning during training corresponds to proper learning.

CAPRI^2^ captures crucial elements of the development of mental representations as well as goal prediction in infants. These building blocks and inference mechanisms allowed us to model the developmental pathway of goal-predictive gaze shifts in infants between 6 and 18 months of age. We conclude that the modeled event-predictive representations and the combination of short-term retrospective, instantaneous, and active inference mechanisms, which strive to both maximize interpretation coherence and minimize anticipated uncertainty, capture key elements of physical reasoning and action understanding in human cognition.

## Materials and methods

In the following sections, implementation, formal information processing, and the training and testing procedures of CAPRI^2^ are detailed.

### Learning event models

The model learns about four events (*e*_still_, *e*_random_, *e*_reach_, *e*_transport_, see below) via supervised training. Each event shows two objects (potential actor and patient) and is composed of a start condition, unfolding dynamics, and an end condition. For each component of an event, a separate neural network module is trained that predicts the mean and the variance of the sensory observations *o*(*t*) at time *t* by means of a multivariate Gaussian. The prediction depends on the chosen gaze policy *π*(*t* − 1), and, within an event, also on the previous observation *o*(*t* − 1). The predictions of the event components are represented as follows:

- The start condition is modeled by $P_{e_{i}}^{start}\left[o\left(t\right)|\pi \left(t-1\right)\right]$. This could, for example, represent a hand starting to move towards a distant object.
- The event dynamics are modeled by $P_{e_{i}}^{event}\left[o\left(t\right)|o\left(t-1\right),\pi \left(t-1\right)\right]$: When the hand, for example, keeps on moving towards the object.
- The prediction of the end condition is modeled by $P_{e_{i}}^{end}\left[o\left(t\right)|o\left(t-\kappa \right),\pi \left(t-1\right)\right]$ with *κ* representing the retrospective time horizon of the ongoing event *e*_*i*_, that is, 1 ≤ *κ* ≤ *τ*′, where *τ*′ denotes the elapsed time since the previous event transition or the beginning of the current sequence. The prediction may characterize, for example, that the moving hand finally grasps the object.

### Predicting event probabilities and model inference

CAPRI uses the learned likelihoods for each event *e*_*i*_ to calculate the event probability (*P*[*e*_*i*_(*t*)∣*O*(*t*), Π(*t* − 1)]) given the previous observations *O*(*t*) = (*o*(*t*), *o*(*t* − 1), …, *o*(1)) and policies Π(*t* − 1) = (*π*(*t* − 1), *π*(*t* − 2), …*π*(0)) at each point in time *t*. The model considers three situations:

- A new event sequence starts. This refers to the start condition of event *e*_*i*_:
  $$\begin{array}{c} P\left[o\left(1\right)|\pi \left(0\right)\right]=P_{e_{i}}^{start}\left[o\left(1\right)|\pi \left(0\right)\right]. \end{array}$$ (1)
- The event remains the same (*e*_*i*_(*t*) = *e*_*j*_(*t* − 1)). This refers to the dynamics of event *e*_*i*_:
  $$\begin{array}{c} P\left[o\left(t\right)|o\left(t-1\right),\pi \left(t-1\right),e_{i}\left(t\right),e_{i}\left(t-1\right)\right]=P_{e_{i}}^{event}\left[o\left(t\right)|o\left(t-1\right),\pi \left(t-1\right)\right]. \end{array}$$ (2)
- The event changes (*e*_*i*_(*t*)≠*e*_*j*_(*t* − 1)). This refers to the end condition of the previous event *e*_*j*_ and the start condition of the new event *e*_*i*_:
  $$\begin{array}{c} \begin{array}{c} P[o\left(t\right)|o\left(t-1\right),\pi \left(t-1\right),e_{i}\left(t\right),e_{j}\left(t-1\right)]=\\ P_{e_{i}}^{start}\left[o\left(t\right)|\pi \left(t-1\right)\right]\cdot P_{e_{j}}^{end}\left[o\left(t\right)|o\left(t-1\right),\pi \left(t-1\right)\right]. \end{array} \end{array}$$ (3)

The model calculates the probabilities of each event by:

$$\begin{array}{c} \begin{array}{c} P[e_{i}\left(t\right)|o\left(t\right),o\left(t-1\right),\pi \left(t-1\right),e_{j}\left(t-1\right)]=\\ \frac{P\left[o\left(t\right)|o\left(t-1\right),\pi \left(t-1\right),e_{i}\left(t\right),e_{j}\left(t-1\right)\right]\cdot P\left[e_{i}\left(t\right)|e_{j}\left(t-1\right)\right]}{\sum_{e_{h}}P\left[o\left(t\right)|o\left(t-1\right),\pi \left(t-1\right),e_{h}\left(t\right),e_{j}\left(t-1\right)\right]\cdot P\left[e_{h}\left(t\right)|e_{j}\left(t-1\right)\right]}, \end{array} \end{array}$$ (4)

where assume a high prior probability of no event transition, i.e., *P*[*e*_*i*_(*t*)∣*e*_*j*_(*t* − 1)] = .9 for *i* = *j*. That is, the implementation assumes that the event will stay the same with a probability of 90%.

### Actively inferring gaze positions

The model can actively influence the observation it gets by choosing one of three gaze policies: fixating the actor (*π*_actor_), the patient (*π*_patient_), or neither. The location of fixation influences the standard deviation *σ* of the normally distributed noise applied to the sensory information related to the actor and/or patient. We added noise with *σ* = 0.001 for the currently fixated object (actor/patient) and with *σ* = 0.1 for the other object. To maximize the accuracy of its predictions, the model chooses the gaze policy that minimizes surprise and, therefore, the expected free energy using

$$\begin{array}{c} \pi \left(t\right)=\text{arg}\underset{\pi_{i}}{\text{min}\;}\hat{FE}\left(\pi_{i},t\right) \end{array}$$ (5)

Gumbsch and colleagues [50] used the conceptualization of expected free energy of Friston et al. [65]. Because they controlled the attractiveness of the visual stimuli, the distribution of the desired states was assumed to be approximately uniform in the modeled experiments. Hence they focused only on the predicted uncertainty:

$$\begin{array}{c} \hat{FE}\left(\pi ,t\right)=E_{t\leq t^{\prime }\leq \tau ,e_{i}}[H[P\left(o\left(t^{\prime }\right)|e_{i}\left(t^{\prime }\right),\pi \right)]], \end{array}$$ (6)

where the number of future event boundaries considered for the free-energy calculation is specified by *τ*. For *τ* = 0, for example, no event boundary would be considered, so the model tries to minimize the free energy of the currently unfolding event, i.e. by fixating the actor (gaze policy *π*_actor_) during *e*_reach_. For *τ* = 1, the model takes the next event boundary into account. In the case of a reaching movement, this means that the grasp (actor contacting the patient) is considered because the event changes from *e*_reach_ to *e*_transport_. Thus, the free energy can be reduced by fixating the patient (gaze policy *π*_patient_), as the currently unfolding reach event will end in this location [50, CAPRI also used τ = 1]. The chosen gaze policy influences the next observation because it reduces the noise applied to the sensory information about the fixated object. For larger *τ*, more future event boundaries may be considered. In this case, however, the model’s gaze behavior became more erratic early on.

### Optimizing coherence

CAPRI^2^ extends the original CAPRI model by a mechanism to update its agency estimations, simulating humans who try to maximize coherence of their perception and interpretation [27–32]. The expected likelihood of an observation given earlier observations *O*(*t* − 1) and gaze policies Π(*t* − 1) represents the coherence of a movement sequence. This likelihood is given by

$$\begin{array}{c} \begin{array}{c} P\left[o\left(t\right)|O\left(t-1\right),\Pi \left(t-1\right)\right]=\sum_{e_{j}}\sum_{e_{i}}\;P\left[o\left(t\right)|e_{i}\left(t\right),e_{j}\left(t-1\right),o\left(t-1\right),\pi \left(t-1\right)\right]\;\cdot \\ P\left[e_{i}\left(t\right)|e_{j}\left(t-1\right),o\left(t\right),o\left(t-1\right),\pi \left(t-1\right)\right]\;\cdot P\left[e_{j}\left(t-1\right)|O\left(t-2\right),\Pi \left(t-2\right)\right] \end{array}. \end{array}$$ (7)

The agency estimation *ae*_*a*_ of the actor *a* that maximizes this likelihood also maximizes the log-likelihood. It is used to optimize the coherence of the interpretation of a sequence, taking the negative log-likelihood as a loss. This adaptation loss is given by

$$\begin{array}{c} \begin{array}{cc} L_{ae} & =-\sum_{t}\;\text{log}(P[o(t)|O(t-1),\Pi (t-1)])\\ & =-\text{log}(P[o(1)|\Pi (0)])-\sum_{t=2}^{\tau }\;\text{log}(P[o(t)|o(t-1),\Pi (t-1)]), \end{array} \end{array}$$ (8)

for an event sequence length *τ*. When testing a new actor after a given training phase, the model applies the initial agency estimation and the patient shape and then updates these values to calculate the posterior agency estimation after each trial during testing. The log-likelihood and, hence, the coherence are optimized by adapting the agency estimation *ae*_*a*_ of the actor after seeing a complete sequence, using gradient descent. The adaptation can be formalized as:

$$\begin{array}{c} \Delta ae_{a}=-\eta \cdot \frac{\partial (-\sum_{t}\;\text{log}(P[o(t)|O(t-1),\Pi (t-1)]))}{\partial \;ae_{a}}, \end{array}$$ (9)

$$\begin{array}{c} ae_{a}\leftarrow ae_{a}+\Delta ae_{a}, \end{array}$$ (10)

with the learning rate *η*, the derivative ∂, and the adaptation of the agency estimate Δ*ae*_*a*_. This adaptation models an infant who sees an action and tries to maximize coherence by adapting the agency estimation of the actor in order to maximize the likelihood of seeing each observation, given earlier observations and previous gaze behavior (e.g., tracking the moving actor or shifting the gaze to the goal object).

### Simulation Setup in CAPRI^2^

#### Observations

**Fig 8:**
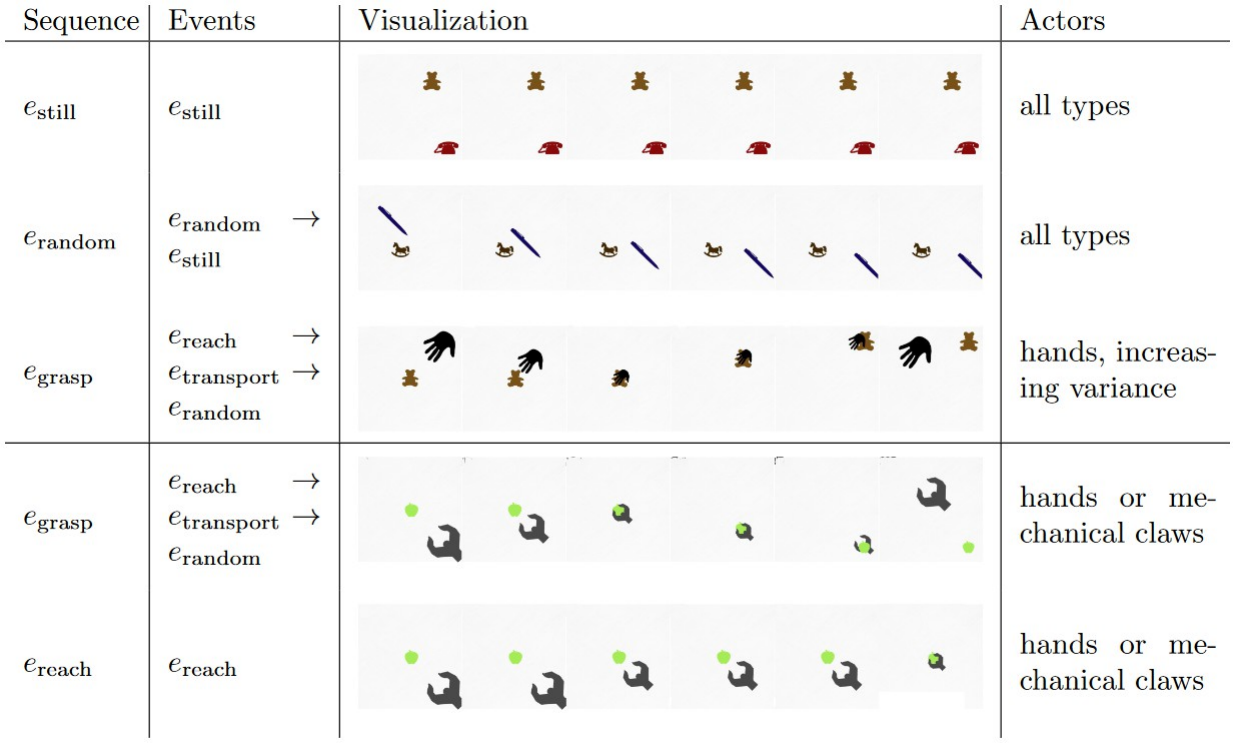
Sequences and events used for training and testing. The Figure is adapted from Gumbsch et al. [50, Fig 4]. Event sequences consisted of one to three events. The top three event sequences were used for training. The model was tested with various actor shapes (varying from 0 ‘hand-like’ to 1 ‘claw-like in steps of 0.1) and with full grasping sequences with action effect as well as reach-only sequences without action effect. The length of the sequences, i.e. the number of timesteps, varied as a function of the included events.

Each observation shows two entities (potential actor and patient). All sequences presented to the model are based on four events as depicted in Fig 8:

- A ‘standing still’ event (*e*_still_) shows both objects remaining motionless.
- A ‘random motion’ event (*e*_random_) shows one entity (the actor) moving in a random fixed direction while the other object (the patient) stays still.
- A ‘reaching’ event (*e*_reach_) shows one entity (the actor) acting like a grasping hand: The actor moves towards the other object (the patient) until both objects are in contact.
- A ‘transport’ event (*e*_transport_) can occur after *e*_reach_ and shows both entities moving together with a random constant velocity to a randomly set goal location.

Three event sequences occurred in training: ‘still’ consisting of only *e*_still_, ‘random motion’ including *e*_random_ followed by *e*_still_, and ‘full grasping with action effect’ including *e*_reach_ followed by *e*_transport_ and *e*_random_, showing a hand (or another actor, varying in shape between hand and claw) reaching for and grasping an object, transporting it, and then moving away. Event sequences lasted between 100 and 270 time steps. For testing, we used sequences performed by actors varying in shape between hand and claw: full grasping with action effect (i.e., *e*_reach_, *e*_transport_, *e*_random_) and reaching without action effect, consisting of only *e*_reach_.

Observations from these sequences were forwarded to the model at each time step. An observation *o*(*t*) consists of an 18-dimensional real-valued vector that encodes various attributes of agent and patient (denoted by *a* and *p*, respectively):

- the positions (*x*^*a*^, *x*^*p*^) and velocities (*v*^*a*^, *v*^*p*^) of actor and patient in three dimensions,
- the position of the actor relative to the patient in three dimensions (*x*^*p*−*a*^ = *x*^*p*^ − *x*^*a*^),
- the Euclidean distance between the actor and patient (*d*^*a*, *p*^),
- the agency estimation of the actor (initially based on its shape) and the shape of the patient (one-dimensional: *ae*_*a*_ and *s*_*p*_).

In this vector, the values describing the actor and the patient are position encoded, so the challenge of assigning actor and patient roles is sidestepped.

The agency estimation in CAPRI^2^ replaced the shape of the actor used in the previous CAPRI model [50]. Agency cues (i.e., movements and action effects) are crucial because they inform the model about the potential events typically executed by actors of various shapes. In CAPRI^2^, *ae*_*a*_ = 0.2 corresponds to a canonical hand and *ae*_*a*_ = 0.8 to a mechanical claw.

#### Training and testing

Training attempted to simulate everyday experiences infants may gain. After each training phase, the model received test trials intended to mimic experimental conditions from developmental studies in which infants watched videos of a hand or claw reaching for and grasping a goal object [43, 45]. We assume that early in development, infants mostly observe reaching events done by hands, e.g. from their caretakers’, or, later, their own. As they grow older, infants learn that other entities also perform similar motions, e.g., seeing that a fork can “reach” for food. To simulate this development during CAPRI^2^’s training, grasping sequences were initially only performed by hands (shape *μ*_*ae*_ = 0.2, *σ*_*ae*_ = 0.1), but the variance of actor shapes increased over training phases towards a uniform distribution between *ae*_*a*_ = 0 ‘hand-like’ and *ae*_*a*_ = 1 ‘claw-like’.

The training included 30 training phases consisting of 100 event sequences drawn uniformly from ‘still’, ‘random motion’, and ‘full grasping with action effect’ (Fig 8). For each sequence, the model chose a random fixed gaze policy. The parameters of the models were adapted after each sequence.

During testing, CAPRI^2^ observed actors with various shapes. We specifically investigated hand-like shapes with *μ*_*ae*_ = 0.4 (similar but slightly different to hands seen in training, as in the hand of a stranger). In the test trials, both hand and claw shapes could perform grasping movements. Each test phase included 12 trials for each actor, who performed either ‘full grasping with action effect’ or ‘only-reach without action effect’ sequences (Fig 8). For CAPRI^2^’s coherence-optimizing mechanism, the agency estimate was set initially to the shape of the actor tested, e.g. *ae*_*a*_ = 0.4, and then adapted after each trial using stochastic gradient descent with a learning rate of 10^−5^ and a momentum rate of 0.3 for five update steps. Overall, we trained and tested twenty models using different training seeds. (The results in Figs 5–6 report the performance averaged over the twenty models).

Similarly to developmental studies [45], we analyzed how action effects, e.g., observing the actor lifting the patient after its grasp, affect the model’s gaze behavior. Thus, we compared different grasping sequences and tested the model using full grasping sequences as well as shorter grasping sequences without action effects, which are cut off after *e*_reach_.
